# Real-world bleeding in patients with acute coronary syndrome (ACS) undergoing percutaneous coronary intervention (PCI) and prescribed different combinations of dual antiplatelet therapy (DAPT) in England: a population-based cohort study emulating a ‘target trial’

**DOI:** 10.1136/openhrt-2022-001999

**Published:** 2022-08-12

**Authors:** Maria Pufulete, Jessica Harris, Koen Pouwels, Barney C Reeves, Daniel Lasserson, Yoon K Loke, Andrew Mumford, Kalaivani Mahadevan, Thomas W Johnson

**Affiliations:** 1 Bristol Trials Centre, Bristol Medical School, University of Bristol, Bristol, UK; 2 Bristol Heart Institute, University of Bristol, Bristol, UK; 3 Health Economics Research Centre, Nuffield Department of Population Health, University of Oxford, Oxford, UK; 4 Warwick Medical School, University of Warwick, Coventry, UK; 5 Department of Geratology/AGM, Oxford University Hospitals NHS Foundation Trust, Oxford, UK; 6 Norwich Medical School, University of East Anglia, Norfolk, UK; 7 School of Cellular and Molecular Medicine, Bristol Medical School, University of Bristol, Bristol, UK

**Keywords:** percutaneous coronary intervention, electronic health records, acute coronary syndrome, epidemiology, pharmacology, clinical

## Abstract

**Objective:**

To estimate the incidence and HRs for bleeding for different dual antiplatelet therapies (DAPT) in a real-world population with acute coronary syndrome (ACS) undergoing percutaneous coronary intervention (PCI) in England.

**Design:**

A retrospective, population-based cohort study emulating a target randomised controlled trial (tRCT).

**Data sources:**

Linked Clinical Practice Research Datalink (CPRD) and Hospital Episode Statistics (HES).

**Setting:**

Primary and secondary care.

**Participants:**

Patients ≥18 years old with ACS undergoing emergency PCI.

**Interventions:**

Aspirin and clopidogrel (AC, reference) versus aspirin and prasugrel (AP) or aspirin and ticagrelor (AT); AP evaluated only in patients with ST-elevation myocardial infarction (STEMI).

**Main outcome measures:**

Primary: any bleeding up to 12 months after the index event (HES- or CPRD- recorded). Secondary: HES-recorded bleeding, CPRD-recorded bleeding, all-cause and cardiovascular mortality, mortality from bleeding, myocardial infarction, stroke, additional coronary intervention and major adverse cardiovascular and cerebrovascular events (MACCE).

**Results:**

In ACS, the rates of any bleeding for AC and AT were 89 per 1000 person years and 134 per 1000 person years, respectively. In STEMI, rates for AC, AP and AT were 93 per 1000 person years, 138 per 1000 person years and 143 per 100 person years, respectively. In ACS, compared with AC, AT increased the hazard of any bleeding (HR: 1.47, 95% CI 1.19 to 1.82) but did not reduce MACCE (HR: 1.06, 95% CI 0.89 to 1.27). In STEMI, compared with AC, AP and AT increased the hazard of any bleeding (HR: 1.77, 95% CI 1.21 to 2.59 and HR: 1.50, 95% CI 1.10 to 2.05, respectively) but did not reduce MACCE (HR: 1.10, 95% CI 0.80 to 1.51 and HR: 1.21, 95% CI 0.94 to 1.51, respectively). Non-adherence to the prescribed DAPT regimen was 28% in AC (29% in STEMI only), 31% in AP (STEMI only) and 33% in AT (32% in STEMI only).

**Conclusions:**

In a real-world population with ACS, DAPT with ticagrelor or prasugrel are associated with increased bleeding compared with DAPT with clopidogrel.

**Trial registration number:**

ISRCTN76607611.

**WHAT IS ALREADY KNOWN ON THIS TOPIC**

- Randomised controlled trials (RCTs) have shown that in patients with acute coronary syndrome (ACS) undergoing percutaneous coronary intervention (PCI), more potent dual antiplatelet therapy (DAPT) using prasugrel and ticagrelor is more effective at reducing cardiovascular events at the expense of more bleeding events compared with less potent DAPT using clopidogrel.

**WHAT THIS STUDY ADDS**

- In ACS and STEMI-only real-world populations, DAPT using ticagrelor or prasugrel was associated with increased rates of bleeding but no reductions in cardiovascular events.
- In 12 months post ACS, up to one-fifth of patients switched DAPT prescription and up to one-third of patients did not adhere to DAPT, with rates of non-adherence slightly higher in patients taking more potent DAPT (with ticagrelor or prasugrel). These rates are higher than those reported in trials.

**HOW THIS STUDY MIGHT AFFECT RESEARCH, PRACTICE OR POLICY**

- The results of this study should be carefully considered by clinicians and decision-makers alongside RCT evidence when making recommendations about DAPT, given that more potent DAPT may increase risk of bleeding without reducing cardiovascular events.

## Introduction

Contemporary treatment of patients with acute coronary syndrome (ACS), with or without ST-elevation, focuses on an early invasive coronary intervention strategy combined with potent dual antiplatelet therapy (DAPT)—aspirin and ticagrelor for ACS or aspirin and prasugrel for ST-elevation myocardial infarction (STEMI).^1 2^ Two landmark randomised controlled trials (RCTs) conducted over 10 years ago, TRITON and PLATO, shifted prescribing from less potent clopidogrel to more potent prasugrel or ticagrelor,^3 4^ as these reduced the risk of future myocardial infarction (MI) and particularly stent thrombosis.

Real-world use of these potent antiplatelet agents is likely to result in a higher risk of bleeding than that reported in the selectively recruited populations of RCTs.^5 6^ The increased bleeding risk from DAPT with ticagrelor and prasugrel has not been adequately quantified in previous RCTs, which were designed primarily to investigate ischaemic rather than bleeding events. We, therefore, designed a target trial using routinely collected clinical data to emulate a hypothetical RCT,^7^ hereafter referred to a tRCT, to compare the risk of bleeding for DAPT using prasugrel or ticagrelor with DAPT using clopidogrel. We used the framework recommended by the Cochrane Bias and Non-randomised Studies Methods Groups^8^ to define the appropriate patient population, treatment assignment, specification of ‘time zero’, outcomes and follow-up.

## Methods

### Data sources

Clinical Practice Research Datalink (CPRD) is a database of primary care electronic health record data covering roughly 7% of the UK population.^9^ CPRD is linked with Hospital Episode Statistics (HES), which covers hospital admissions for all English patients whose treatment is funded by the UK National Health Service.^10^ The study protocol was approved by the Independent Scientific Advisory Committee of the CPRD (protocol number: 16_126R).^11^

### Study population

We specified a tRCT for patients with ACS undergoing emergency percutaneous coronary intervention (PCI). We also identified a subgroup of ACS with STEMI because prasugrel is recommended only for this population.^1 2^ Patients were eligible if they had a PCI with an ACS diagnosis (index event) in the same hospital admission recorded in HES during the study period (1 April 2010–31 January 2017). Full details of eligibility, exclusion criteria and procedure/diagnosis codes for identifying the population for the tRCT are listed in the study protocol.^11^

### Interventions

In ACS, risk of bleeding was compared between aspirin plus clopidogrel (AC, reference) versus aspirin plus ticagrelor (AT). In STEMI, risk of bleeding was compared between AC (reference) versus AT, and between AC versus aspirin plus prasugrel (AP). Because HES data do not contain medication information, we used the first prescription in CPRD, recorded during the first 2 months after discharge from the index event, as a proxy for the medications that patients started in hospital. Patients with no antiplatelet prescription or who experienced a major bleed or major adverse cardiovascular or cerebrovascular event (MACCE) before the first antiplatelet prescription in CPRD within the 2-month window were excluded from the main analysis. A bleed or MACCE would likely lead to the DAPT assigned in hospital being changed after the event.

### Outcomes

The primary outcome was time to the first bleeding event (HES or CPRD). Secondary outcomes were HES-recorded bleeding (requiring hospital admission), CPRD-recorded bleeding, all-cause mortality, cardiovascular mortality, mortality from bleeding, MI, stroke, additional coronary intervention and MACCE, defined as any of MI, stroke, cardiovascular mortality or additional coronary intervention. To ensure that bleeds recorded in CPRD were not duplicating events recoded in HES, CPRD-reported bleeding was defined as any CPRD bleed without any HES bleed recorded within ±14 days of the CPRD bleed.

### Confounding and co-interventions

Confounders and co-interventions were specified a priori through a systematic review, interviews with cardiologists and a survey with additional cardiologists.^11^

### Sample size

We used preliminary feasibility counts provided by CPRD to identify numbers of eligible participants and proportions assigned to different therapies. We estimated rates of any bleeding event expected with the different therapies based on published studies, 9% for AC and 12% for AP and AT.^3 12–14^ These estimates gave an expected number of first bleeding events of at least 700. Assuming a ratio of 8:1 (AC:AP or AC:AT), we estimated that 6738 patients assigned to AC (reference) versus 842 assigned to AP or 770 to AT would allow us to detect HRs of 1.74 with 90% power and 5% statistical significance, assuming a correlation of DAPT treatment with other covariates of 0.5.

### Data cleaning and managing missing data

For smoking status and body mass index (BMI), we used the most recent record in CPRD and applied standard data cleaning rules.^15 16^ For binary variables, we assumed that no recorded event code meant absence of the event. We examined all non-binary variables for missing data. Smoking and BMI had 4% and 8% missing values, respectively; these were replaced with age-adjusted and sex-adjusted averages estimated from the rest of the cohort.

### Statistical analyses

Statistical analyses were undertaken using Stata V.15.1 (StataCorp LLC, College Station, Texas, USA). We used descriptive statistics to summarise the characteristics of the different intervention groups and standardised mean differences to compare them. We estimated hazard rates of any bleeding (number of events/person time, including only time up to the first bleed) with 95% CIs for each group. We censored all bleeds at the General Practice (GP) transfer out date or last collection date, thereby ignoring any bleeds in the HES dataset recorded after this period.

We conducted separate analyses for ACS (AC vs AT) and STEMI (AC vs AP vs AT) emulating an intention-to-treat analysis for the antiplatelet regimens assigned by the first prescription of DAPT in CPRD. We calculated propensity scores (PS) for the assigned interventions using a backward stepwise logistic regression with signiﬁcance level for removal from the model set at 0.25. The AC versus AP versus AT analysis in STEMI was conducted using a multinomial logistic regression due to the three interventions.

All confounders identified were included in these stepwise models. Criteria for excluding tails of PS distributions were decided by reviewing the bleeding events between interventions, based on cut points of the PS at 5th, 25th, 50th, 75th and 95th percentiles.^17^ There was good overlap of PS distributions; therefore, no tails needed to be excluded (online supplemental material). Kaplan-Meier curves were generated after adjusting by the inverse probability of treatment weights using the PS,^18^ where the weights were defined as 1/PS for the treatment assigned.

10.1136/openhrt-2022-001999.supp1 Supplementary data

We used Cox regression models to estimate crude and adjusted HRs with 95% CIs for the time to first event (primary and secondary outcomes), comparing intervention groups for each population. Participants free from a bleeding event were censored at 12 months after the index event. For each analysis, we adjusted for all prespecified confounders and the PS. All continuous variables (calendar year, age, BMI and PS) were included in models as cubic splines with knots set at the 25th and 75th percentiles. Confounders (including the Charlson Comorbidity Index^19^ for mortality) were included using a backward stepwise approach with signiﬁcance level for removal from the model set at 0.25, and additionally adjusted for PS.

### Sensitivity analyses

We prespecified four sensitivity analyses^11^: (1) multiple imputation for unknown intervention group, based on the PS calculated from the main analysis populations,^17^ (2) Including patients at low risk of bleeding (excluding stage 4/stage 5 chronic kidney disease, anaemia, clotting disorder, cancer, liver cirrhosis with portal hypertension, stroke or surgery within the last 30 days), (3) Including patients who had a first HES bleed after the patient had transferred out of a GP practice or after the last collection date for that GP practice and (4) excluding patients who changed medication before first observed bleeding event if >10% changed medication. This was not conducted because only 3% of our population changed medication before their first bleed.

### Subgroup analyses

The following subgroups were investigated: diabetic versus non-diabetic, chronic kidney disease versus non-chronic kidney disease and concurrent prescription for proton pump inhibitor (PPI). For each subgroup, the main primary outcome analysis (adjusted by PS and all selected confounders) was repeated, including a subgroup by intervention interaction term.

### Treatment switches and adherence

Treatment switch/discontinuation was defined as stopping aspirin or the second antiplatelet or starting a different antiplatelet to those assigned at baseline. Stopping aspirin or the second antiplatelet was defined as a gap between repeat prescriptions >1.5 times the number of days’ supply of the last prescription. Starting another antiplatelet was defined as a patient receiving at least one prescription of the antiplatelet during follow-up.

Adherence was defined using the medication possession ratio (MPR).^20^ MPR was calculated as the total number of days of available medication (quantity of drug prescribed divided by the daily dose) divided by the number of days to the end of follow-up (1 year, or to date of death, or to 31 July 2017 for later events). For example, for AC, overall MPR was calculated as the average MPR of aspirin and clopidogrel. Non-adherence was defined as MPR <0.80.

## Results

**Figure 1:**
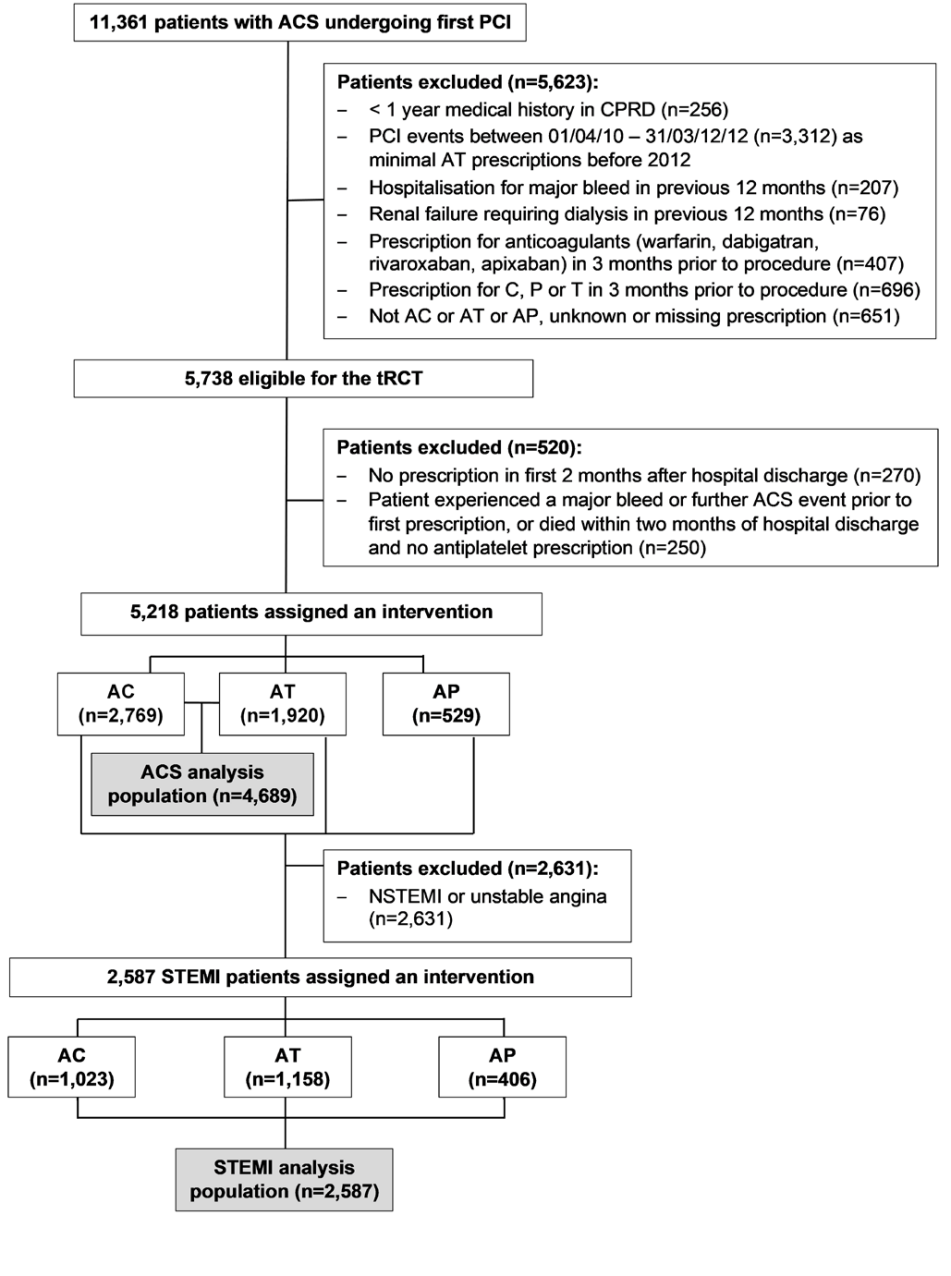
Flow diagram describing the construction of the tRCT. AC, aspirin and clopidogrel; ACS, acute coronary syndrome; AP, aspirin and prasugrel; AT, aspirin and ticagrelor; CPRD, Clinical Practice Research Datalink; PCI, percutaneous coronary intervention; STEMI, ST-elevation myocardial infarction; tRCT, target randomised controlled trial.

Figure 1 shows the construction of tRCT. We included 4689 and 2587 participants and excluded 520 and 306 (10% and 11% of eligible participants) in the ACS and STEMI groups, respectively. The baseline characteristics of included participants are listed in table 1. Generally, compared with patients receiving AC, patients receiving AT or AP were younger and had a higher proportion of men and smokers and fewer comorbidities. In both populations, the median length of hospital stay was similar between different DAPT groups (2 days for ACS and 3 days for STEMI). The baseline characteristics of excluded participants are shown in the online supplemental material.

**Table 1:** Baseline characteristics of ACS and STEMI participants, overall and by intervention status (AC vs AP vs AT)

		ACS				STEMI					
		Overall n=4689	AC n=2769	AT n=1920	SMD* (AC vs AT)	Overall n=2587	AC n=1023	AP n=406	AT n=1158	SMD (AP vs AC)	SMD (AT vs AC)
Demography											
Year of event; n (%)	2012/2013	1303 (28%)	1090 (39%)	213 (11%)	0.78	713 (28%)	425 (42%)	170 (42%)	118 (10%)	0.12	0.91
	2013/2014	1147 (24%)	710 (26%)	437 (23%)		629 (24%)	288 (28%)	98 (24%)	243 (21%)		
	2014/2015	1025 (22%)	493 (18%)	532 (28%)		564 (22%)	163 (16%)	78 (19%)	323 (28%)		
	2015/2016	733 (16%)	302 (11%)	431 (22%)		423 (16%)	99 (10%)	43 (11%)	281 (24%)		
	2016/2017†	481 (10%)	174 (6%)	307 (16%)		258 (10%)	48 (5%)	17 (4%)	193 (17%)		
Age, years (mean; SD)		64.6 (12.4)	66.1 (12.4)	62.5 (11.9)	0.30	63.0 (12.3)	65.8 (12.7)	58.8 (10.0)	62.0 (12.0)	0.61	0.31
Sex; n (%)	Male	3418 (73%)	2007 (72%)	1411 (73%)	0.02	1923 (74%)	736 (72%)	331 (82%)	856 (74%)	0.23	0.04
	Female	1271 (27%)	762 (28%)	509 (27%)		664 (26%)	287 (28%)	75 (18%)	302 (26%)		
BMI,‡ kg/m^2^ (mean; SD)		28.4 (5.2)	28.3 (5.1)	28.4 (5.3)	0.01	28.0 (4.9)	27.8 (4.8)	28.1 (4.6)	28.0 (531)	0.07	0.04
Ethnic group; n (%)	White	4275 (91%)	2520 (91%)	1755 (91%)	0.01	2382 (92%)	944 (92%)	372 (92%)	1066 (92%)	0.02	0.01
	Non-white	414 (9%)	249 (9%)	165 (9%)		205 (8%)	79 (8%)	34 (8%)	92 (8%)		
Smoking category§; n (%)	Ex-smoker	1443 (32%)	891 (33%)	552 (30%)	0.15	722 (29%)	295 (30%)	105 (27%)	322 (29%)	0.27	0.11
	Non-smoker	1699 (38%)	1047 (39%)	652 (36%)		829 (34%)	359 (37%)	107 (28%)	363 (33%)		
	Smoker	1357 (30%)	730 (27%)	627 (34%)		909 (37%)	318 (33%)	177 (46%)	414 (38%)		
Medical history											
History of MI (ever); n (%)		3616 (77%)	2153 (78%)	1463 (76%)	0.03	1941 (75%)	797 (78%)	290 (71%)	854 (74%)	0.14	0.09
History of CABG/PCI (ever); n (%)		1472 (31%)	899 (32%)	573 (30%)	0.03	593 (23%)	243 (24%)	71 (17%)	279 (24%)	0.10	0.05
Bleeding; n (%)		92 (2%)	50 (2%)	42 (2%)	0.03	63 (2%)	24 (2%)	11 (3%)	28 (2%)	0.02	0.01
Previous surgery; n (%)		178 (4%)	126 (5%)	52 (3%)	0.10	75 (3%)	34 (3%)	10 (2%)	31 (3%)	0.05	0.04
History of IHD (ever); n (%)		4084 (87%)	2489 (90%)	1595 (83%)	0.19	2052 (79%)	842 (82%)	305 (75%)	905 (78%)	0.17	0.10
Comorbidities											
Diabetes; n (%)		924 (20%)	568 (21%)	356 (19%)	0.05	397 (15%)	167 (16%)	47 (12%)	183 (16%)	0.14	0.01
Hypertension; n (%)		1857 (40%)	1192 (43%)	665 (35%)	0.17	705 (27%)	306 (30%)	84 (21%)	315 (27%)	0.21	0.05
Hypercholesterolaemia; n (%)		903 (19%)	597 (22%)	306 (16%)	0.15	263 (10%)	126 (12%)	29 (7%)	108 (9%)	0.16	0.10
Peripheral vascular disease; n (%)		198 (4%)	141 (5%)	57 (3%)	0.11	72 (3%)	36 (4%)	10 (2%)	26 (2%)	0.06	0.08
Stroke; n (%)		17 (0.4%)	12 (0.4%)	5 (0.3%)	0.04	11 (0.4%)	6 (1%)	1 (0.2%)	4 (0.3%)	0.05	0.05
Heart failure; n (%)		308 (7%)	193 (7%)	115 (6%)	0.03	155 (6%)	61 (6%)	23 (6%)	71 (6%)	0.004	0.02
Peptic ulcer disease; n (%)		14 (0.3%)	10 (0.4%)	4 (0.2%)	0.03	3 (0.1%)	1 (0.1%)	0	2 (0.2%)	0.04	0.02
Haemodialysis or renal disease; n (%)		196 (4%)	139 (5%)	57 (3%)	0.11	55 (2%)	29 (3%)	5 (1%)	21 (2%)	0.12	0.08
Cancer; n (%)		191 (4%)	134 (5%)	57 (3%)	0.10	80 (3%)	34 (3%)	11 (3%)	35 (3%)	0.04	0.02
Clotting disorder; n (%)		9 (0.2%)	5 (0.2%)	4 (0.2%)	0.001	5 (0.2%)	1 (0.1%)	0	4 (0.3%)	0.04	0.05
Anaemia; n (%)		106 (2%)	80 (3%)	26 (1%)	0.11	25 (1%)	10 (1%)	4 (1%)	11 (1%)	0.001	0.003
Liver cirrhosis; n (%)		1 (0.02%)	0	1 (0.1%)	0	0 (0%)	0	0	0	--	--
Co-interventions											
NSAIDs; n (%)		903 (19%)	552 (20%)	351 (18%)	0.04	450 (17%)	185 (18%)	65 (16%)	200 (17%)	0.06	0.02
Steroids; n (%)		417 (9%)	256 (9%)	161 (8%)	0.03	209 (8%)	87 (9%)	25 (6%)	97 (8%)	0.09	0.01
PPIs; n (%)		1612 (34%)	994 (36%)	618 (32%)	0.08	785 (30%)	318 (31%)	114 (28%)	353 (30%)	0.07	0.01
Anticoagulants; n (%)		23 (0.4%)	17 (1%)	6 (0.3%)	0.04	11 (0.4%)	4 (0.4%)	3 (1%)	4 (0.3%)	0.05	0.01

*Restricted to 2012–2017.

†The number of eligible patients decreased every year between 2010 and 2017 because of the decline in the number of practices in CPRD GOLD over time.

Missing data^:^ ‡407 patients; §212 patients.

AC, aspirin and clopidogrel; ACS, acute coronary syndrome; AP, aspirin and prasugrel; AT, aspirin and ticagrelor; BMI, body mass index; CABG, coronary artery bypass graft; CPRD, Clinical Practice Research Datalink; IHD, ischaemic heart disease; MI, myocardial infarction; NSAIDs, non-steroidal anti-inflammatory drugs; PCI, percutaneous coronary intervention; PPI, proton-pump inhibitor drugs; SMD, standardised mean difference; STEMI, ST-elevation myocardial infarction.

Table 2 shows the rates of HES, CPRD and total bleeds by antiplatelet regimen. Of the 4689 patients with ACS, 415 (9%) experienced at least one bleed; 209/2769 (8%) assigned to AC and 206/1919 (11%) assigned to AT. Of the 2587 patients with STEMI, 259 (10%) experienced at least one bleed; 80/1023 (8%) assigned to AC, 46/406 (11%) assigned to AP and 133/1157 (12%) assigned to AT.

**Table 2:** Hazard rates of HES-recorded bleeding, CPRD-recorded bleeding and total bleeding (HES and CPRD) by antiplatelet regimen in participants with ACS and STEMI. Rates were calculated taking into account time to first bleed only

	ACS						STEMI								
	AC			AT			AC			AP			AT		
	Total bleeds	Person years	Rate per 1000 person years at risk (95% CI)	Total bleeds	Person years	Rate per 1000 person years at risk (95% CI)	Total bleeds	Person years	Rate per 1000 person years at risk (95% CI)	Total bleeds	Person years	Rate per 1000 person years at risk (95% CI)	Total bleeds	Person years	Rate per 1000 person years at risk (95% CI)
HES-recorded bleeding	63	2731	23.1 (18.0 to 29.5)	54	1888	28.6 (21.9 to 37.3)	22	1008	21.8 (14.4 to 33.2)	9	400	22.5 (11.7 to 43.2)	39	1133	34.4 (25.1 to 47.1)
CPRD-recorded bleeding	161	2671	60.3 (51.7 to 70.3)	170	1807	94.1 (81.0 to 109.4)	62	982	63.2 (49.2 to 81.0)	39	381	102.3 (74.8 to 140.1)	106	1089	97.4 (80.5 to 117.8)
CPRD-recorded and HES-recorded bleeding	209	2344	89.2 (77.9 to 102.1)	206	1543	133.5 (116.5 to 153.1)	80	862	92.8 (74.6 to 115.6)	46	334	137.8 (103.2 to 183.9)	133	928	143.4 (121.0 to 170.0)

AC, aspirin and clopidogrel; ACS, acute coronary syndrome; AP, aspirin and prasugrel; AT, aspirin and ticagrelor; CPRD, Clinical Practice Research Datalink; HES, Hospital Episode Statistics; STEMI, ST-elevation myocardial infarction.

In ACS, AT versus AC was associated with a 24% higher unadjusted hazard rate of HES bleeding (29 vs 23 events per 1000 person years) and a 56% higher unadjusted hazard rate of CPRD bleeding (94 vs 60 events per 1000 person years). In STEMI, AT but not AP was associated with a 58% increase in HES bleeding (23 (AP) vs 34 (AT) vs 22 (AC) events per 1000 person years), but both AP and AT resulted in higher hazard rates of CPRD bleeding, a 62% and 54% increase against AC, respectively (102 (AP) vs 97 (AT) vs 63 (AC) events per 1000 person years).

**Figure 2:**
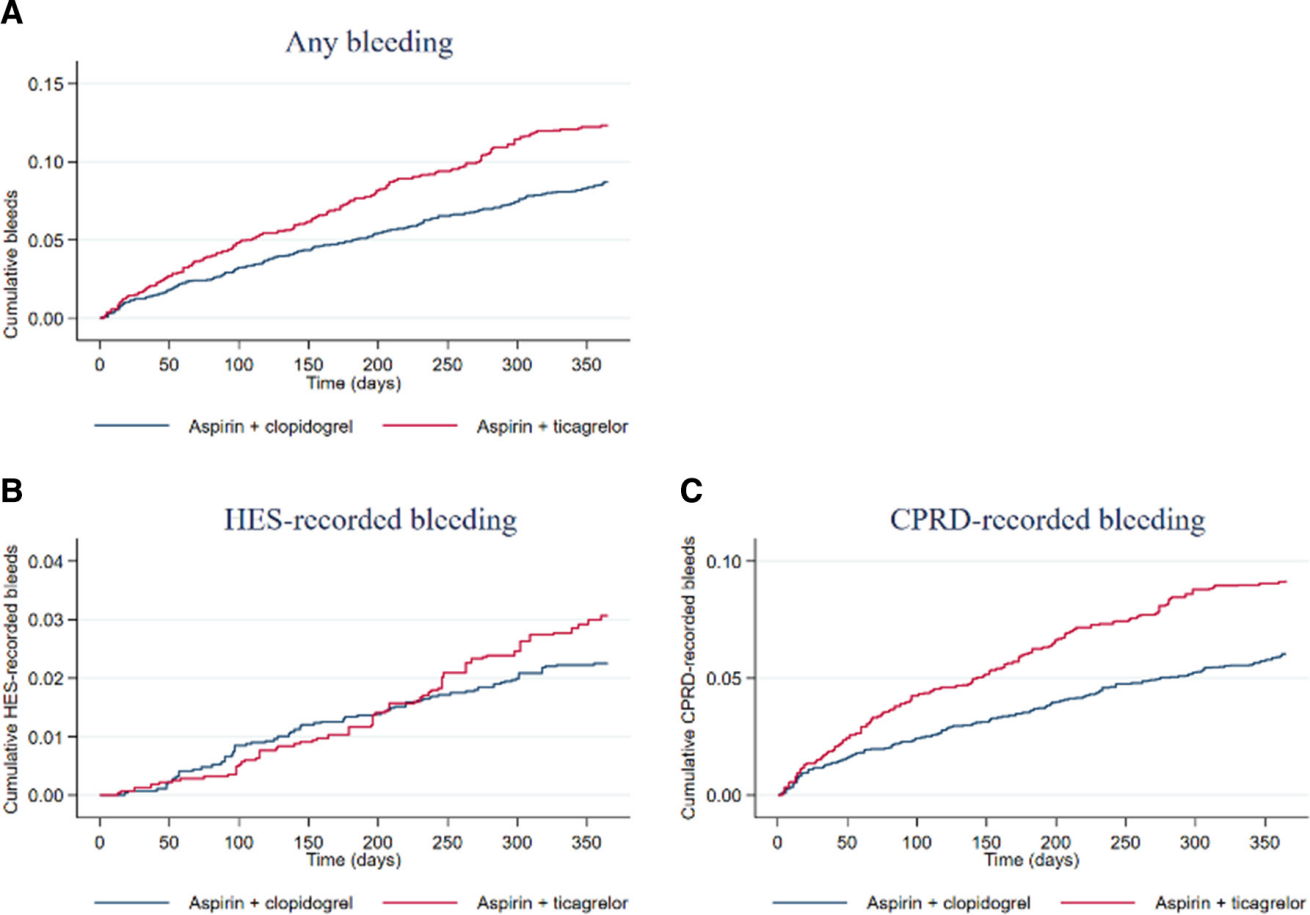
Kaplan-Meier curves displaying cumulative bleeding (any (A), HES-recorded (B) and CPRD-recorded (C)) according to intervention group in the ACS population. Plots are weighted according to the inverse probability of treatment received, and so compare outcomes if all eligible patients received AC or AT. AC, aspirin and clopidogrel; ACS, acute coronary syndrome; AT, aspirin and ticagrelor; CPRD, Clinical Practice Research Datalink; HES, Hospital Episode Statistics.

**Figure 3:**
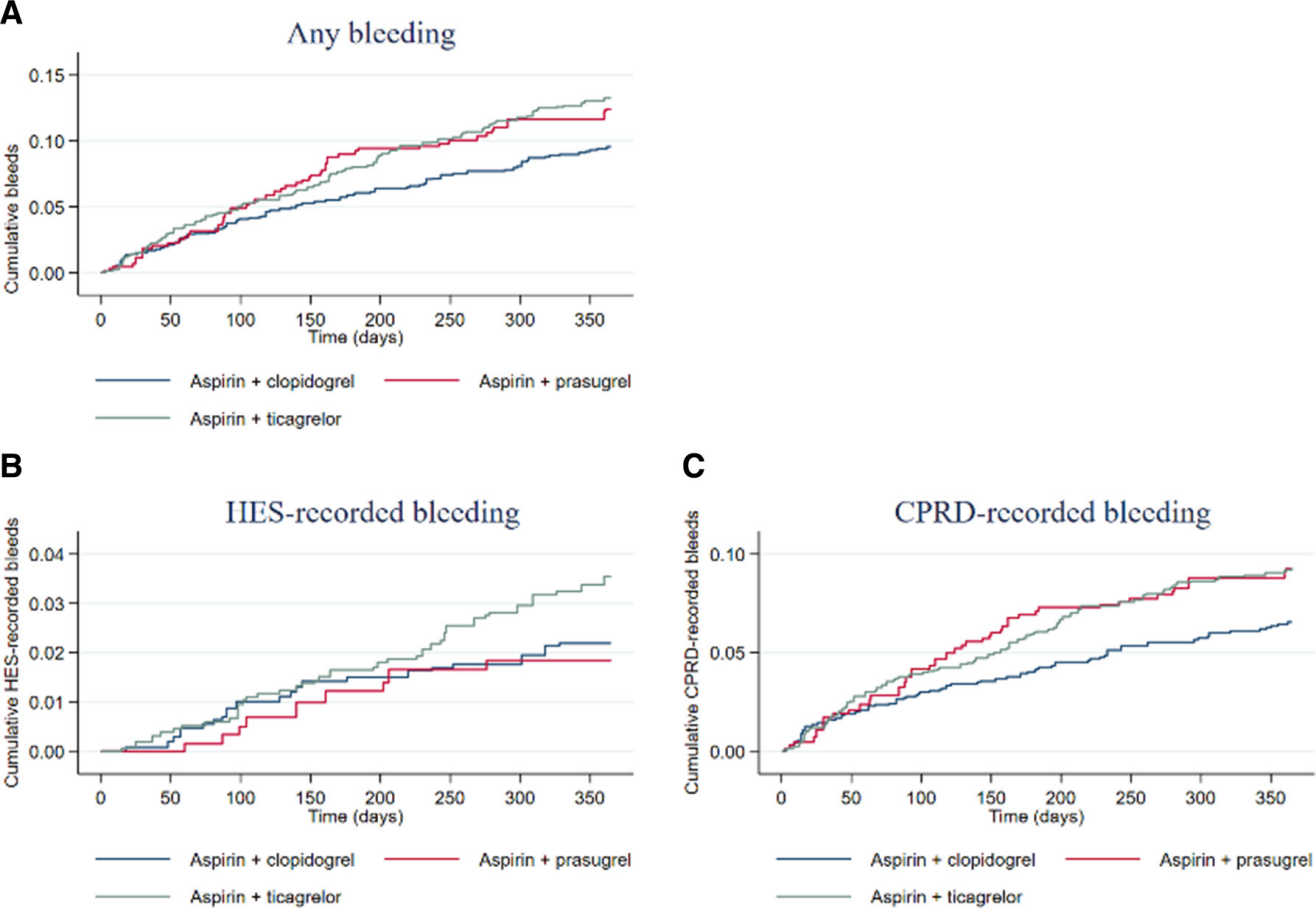
Kaplan-Meier curves displaying cumulative bleeding (any (A), HES-recorded (B) and CPRD-recorded (C)) according to intervention group in the STEMI population. Plots are weighted according to the inverse probability of treatment received, and so compare outcomes if all eligible patients received AC, AP or AT. AC, aspirin and clopidogrel; ACS, acute coronary syndrome; AP, aspirin and prasugrel; AT, aspirin and ticagrelor; CPRD, Clinical Practice Research Datalink; HES, Hospital Episode Statistics; STEMI, ST-elevation myocardial infarction.

The cumulative bleeding events are shown in figures 2 and 3. In both ACS and STEMI, the cumulative hazard of any bleeding was higher in AP and AT than in AC, but these were driven largely by CPRD rather than HES bleeding. In STEMI, there was a lower cumulative hazard of HES bleeding in AP than in AT.

**Figure 4:**
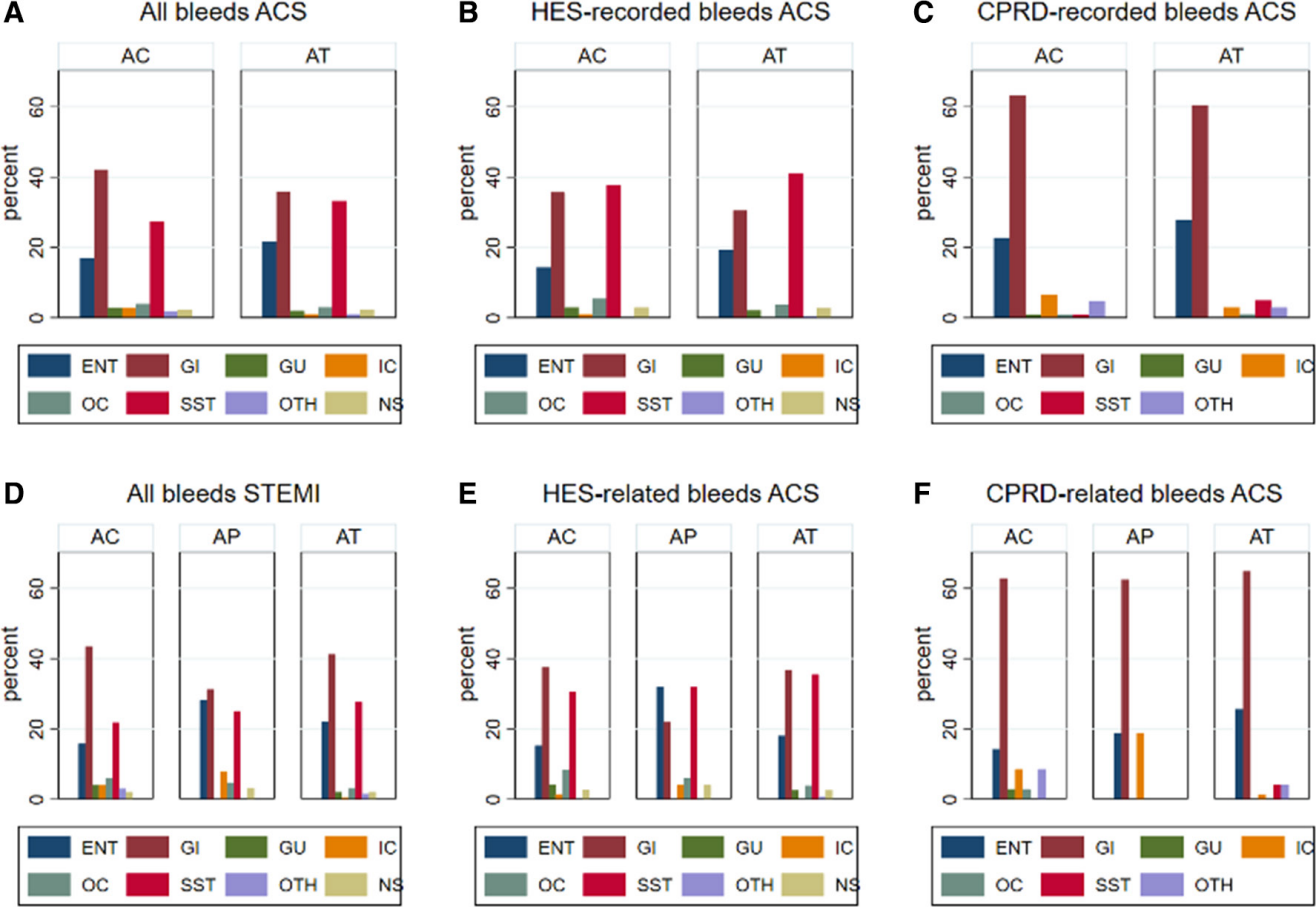
Total bleeding (HES and CPRD), HES-recorded bleeding, CPRD-recorded bleeding by antiplatelet regimen in the ACS and STEMI populations. AC, aspirin and clopidogrel; ACS, acute coronary syndrome; AP, aspirin and prasugrel; AT, aspirin and ticagrelor; CPRD, Clinical Practice Research Datalink; HES, Hospital Episode Statistics; STEMI, ST-elevation myocardial infarction.

Of patients who experienced bleeding, the majority (ACS: 75%; STEMI: 72%) experienced one bleed, about one-fifth (ACS: 19%; STEMI: 22%) experienced two bleeds and the remainder (ACS: 7%; STEMI: 6%) experienced three or more bleeds. Bleeds by site are shown in figure 4. In ACS, there were no major differences in bleeds by site between AC and AT, while in STEMI there were slightly higher numbers of ear, nose and throat bleeds in AP and higher gastrointestinal bleeds in AC. HES bleeding was most commonly gastrointestinal, whereas CPRD bleeding was more frequently skin or soft tissue bleeding.

The unadjusted and adjusted HRs for the primary outcome of any bleeding and secondary outcomes are listed in table 3. After adjustment, AT versus AC significantly increased the hazard of any bleeding (by about 50%) in both ACS and STEMI. In STEMI, AP versus AC increased the hazard of any bleeding by 75%. The HRs remained unchanged following all the sensitivity analyses (see online supplemental material).

**Table 3:** Crude and adjusted HRs for association of antiplatelet prescription (AC vs AP and AC vs AT) with bleeding and ischaemic events and mortality in ACS and STEMI-only patients (2012–2017)

	ACS				STEMI				
		Number of bleeds				Number of bleeds			
	All n=4689	AC n=2769	AT n=1920	HR (95% CI) (AC vs AT)	All n=2587	AC n=1023	AP n=406	AT n=1158	HR (95% CI) (AP vs AC and AT vs AC)
Primary outcome (any bleeding)									
Unadjusted; n (%)	416 (8.9%)	209 (7.5%)	207 (10.8%)	1.48 (1.22 to 1.80)	260 (10.1%)	80 (7.8%)	46 (11.3%)	134 (11.6%)	AP: 1.48 (1.02 to 2.12) AT: 1.53 (1.16 to 2.01)
Adjusted				1.47 (1.19 to 1.82)					AP: 1.77 (1.21 to 2.59)^11^ AT: 1.50 (1.10 to 2.05)^11^
Secondary outcomes (adjusted)									
Major bleeding (HES-recorded); n (%)	117 (2.5%)	63 (2.3%)	54 (2.8%)	1.33 (0.89 to 1.99)	70 (2.7%)	22 (2.2%)	9 (2.2%)	39 (3.4%)	AP: 1.26 (0.56 to 2.87)^12^ AT: 1.99 (1.11 to 3.57)^12^
Minor bleeding (CPRD-recorded); n (%)	332 (7.1%)	161 (5.8%)	171 (8.9%)	1.60 (1.26 to 2.03)	208 (8.0%)	62 (6.1%)	39 (9.6%)	107 (9.2%)	AP: 1.90 (1.26 to 2.88)^13^ AT: 1.53 (1.09 to 2.15)^13^
All-cause mortality; n (%)	104 (2.2%)	70 (2.5%)	34 (1.8%)	0.94 (0.60 to 1.47)	60 (2.3%)	31 (3.0%)	7 (1.7%)	22 (1.9%)	AP: 1.30 (0.54 to 3.17)^14^ AT: 0.92 (0.51 to 1.67)^14^
Cardiovascular mortality; n (%)	48 (1.0%)	32 (1.2%)	16 (0.8%)	0.92 (0.48 to 1.78)	27 (1.0%)	15 (1.5%)	4 (1.0%)	8 (0.7%)	AP: 1.48 (0.45 to 4.87)^15^ AT: 0.60 (0.24 to 1.52)^15^
Mortality from bleeding; n (%)	7 (0.1%)	6 (0.2%)	1 (0.1%)	0.33 (0.04 to 2.86)	6 (0.2%)	4 (0.4%)	2 (0.5%)	0	AP: 2.64 (0.32 to 21.86)^16^ AT: --
MI; n (%)	133 (2.8%)	85 (3.1%)	48 (2.5%)	0.91 (0.61 to 1.34)	72 (2.8%)	27 (2.6%)	12 (3.0%)	33 (2.8%)	AP: 1.20 (0.60 to 2.42)^17^ AT: 1.22 (0.69 to 2.14)^17^
Stroke; n (%)	9 (0.2%)	5 (0.2%)	4 (0.2%)	1.56 (0.40 to 6.03)	5 (0.2%)	2 (0.2%)	1 (0.2%)	2 (0.2%)	AP: 0.71 (0.03 to 16.21)^18^ AT: 0.83 (0.06 to 11.21)^18^
Additional coronary intervention; n (%)	475 (10.1%)	272 (9.8%)	203 (10.6%)	1.03 (0.85 to 1.26)	293 (11.3%)	109 (10.7%)	52 (12.8%)	132 (11.4%)	AP: 1.14 (0.82 to 1.61)^19^ AT: 1.17 (0.88 to 1.55)^19^
MACCE; n (%)	586 (12.5%)	337 (12.2%)	249 (13.0%)	1.06 (0.89 to 1.27)	350 (13.5%)	132 (12.9%)	57 (14.0%)	161 (13.9%)	AP: 1.10 (0.80 to 1.51)^20^ AT: 1.21 (0.94 to 1.51)^20^

Adjusted models included: PS (including year, age, gender, anticoagulants, ischaemic heart disease (ever), hypertension, MI (ever), heart failure, STEMI only and previous surgery (ACS); and including year, age, gender, MI (ever), previous CABG/PCI and hypercholesterolaemia (STEMI)) and also adjusted for the following confounders which remained in the model after backwards elimination: age, gender, BMI, ethnic group, smoking category, previous MI, previous CABG/PCI, previous bleed, previous surgery, previous ischaemic heart disease, diabetes, hypertension, hypercholesterolaemia, peripheral vascular disease, stroke, heart failure, peptic ulcer disease, haemodialysis or renal disease, cancer, clotting disorder, anaemia, liver cirrhosis, NSAIDs, steroids, PPIs, anticoagulants and Charlson Comorbidity Index.

AC, aspirin and clopidogrel; ACS, acute coronary syndrome; AP, aspirin plus prasugrel; AT, aspirin plus ticagrelor; BMI, body mass index; CABG, coronary artery bypass graft; CPRD, Clinical Practice Research Datalink; HES, Hospital Episode Statistics; MACCE, major adverse cardiovascular and cerebrovascular events; MI, myocardial infarction; NSAIDs, non-steroidal anti-inflammatory drugs; PCI, percutaneous coronary intervention; PPIs, proton pump inhibitors; PS, propensity score; SA, sensitivity analysis; STEMI, ST-elevation myocardial infarction.

In ACS, AT increased the hazard of CPRD (by 33%) but not HES bleeding. In STEMI, AT increased the hazard of both HES and CPRD bleeding (twofold and 53%, respectively), while AP increased the hazard of CPRD (by almost twofold) but not HES bleeding. There was no evidence of any subgroup effects. There was no association between antiplatelet prescription and any of the secondary outcomes (table 3, see the online supplemental material for the Kaplan-Meier curves).

Table 4 shows treatment switches. In ACS, there were more switches in patients assigned to AT (404/1920, 21%) than AC (379/2769,14%). In STEMI, the proportion switching was similar for AC and AP (141/1023, 14%; and 60/406, 15%, respectively) but higher for AT (242/1158, 21%). In both populations, between 16% and 20% of patients who switched had a bleed or ischaemic event, with most of these occurring before the switch. Across all intervention groups, ischaemic events were higher in those who switched compared with event rates in the populations overall. Adherence was 72% in AC and 67% in the AT in ACS and 71% in AC, 69% in the AP and 68% in AT in STEMI.

**Table 4:** Treatment switches in the ACS and STEMI-only populations by intervention group (AC and AT) and by type of switch and whether the switch occurred before or after a bleeding or ischaemic event

	Type of switch	Median (IQR) time to switch (months)	Bleed occurred		Ischaemic event^*^ occurred			No ischaemic or bleeding events
			Before switch*	After switch	Before switch (within 2 months)	Before switch	After switch	
		**ACS**						
AC	Discontinued Asp 300/2769 (11%)	8.0 (5.6, 10.9)	19 (6%)	8 (3%)	5 (2%)	19 (6%)	6 (2%)	251 (84%)
	Discontinued C 124/2769 (4%)	8.0 (5.9, 10.2)	8 (6%)	2 (2%)	3 (2%)	12 (10%)	2 (2%)	102 (82%)
	Discontinued AC 84/2769 (3%)	7.9 (5.5, 9.9)	5 (6%)	2 (2%)	3 (4%)	8 (10%)	4 (5%)	66 (79%)
	Initiated a different P2Y12 inhibitor 52/2769 (2%)	2.0 (1.0, 3.8)	2 (4%)	4 (8%)	11 (21%)	11 (21%)	3 (6%)	34 (65%)
AT	Discontinued Asp 210/1920 (11%)	8.0 (6.0, 10.3)	22 (10%)	2 (1%)	1 (1%)	8 (4%)	3 (1%)	177 (84%)
	Discontinued T 154/1920 (8%)	8.1 (6.3, 10.3)	12 (8%)	4 (3%)	2 (1%)	8 (5%)	3 (2%)	129 (84%)
	Discontinued AT 85/1920 (4%)	7.6 (6.1, 9.7)	7 (8%)	3 (4%)	2 (2%)	5 (6%)	2 (2%)	69 (81%)
	Initiated a different P2Y12 inhibitor 151/1920 (8%)	3.3 (1.9, 6.0)	11 (7%)	7 (5%)	2 (1%)	4 (3%)	2 (1%)	128 (85%)
		**STEMI**						
AC	Discontinued Asp 114/1023 (11%)	7.9 (5.6, 11.2)	11 (10%)	0	1 (1%)	6 (5%)	2 (2%)	96 (84%)
	Discontinued C 43/1023 (4%)	7.9 (6.4, 9.5)	7 (16%)	0	1 (2%)	6 (14%)	0/43	32 (74%)
	Discontinued AC 30/1023 (3%)	7.2 (5.1, 9.1)	5 (17%)	0	1 (3%)	3 (10%)	1 (3%)	22 (73%)
	Initiated a different P2Y12 inhibitor 18/1023 (2%)	1.2 (0.8, 3.1)	2 (11%)	1 (6%)	2 (11%)	2 (11%)	1 (6%)	13 (72%)
AP	Discontinued Asp 38/406 (9%)	8.7 (6.4, 10.9)	4 (11%)	0	1 (3%)	1 (3%)	1 (3%)	32 (84%)
	Discontinued P 16/406 (4%)	9.9 (7.9, 11.6)	2 (13%)	0	1 (6%)	2 (13%)	0	12 (75%)
	Discontinued AP 14/406 (3%)	8.8 (6.3, 11.3)	1 (7%)	0	1 (7%)	1 (7%)	1 (7%)	11 (80%)
	Initiated a different P2Y12 inhibitor 22/406 (5%)	2.9 (1.5, 4.6)	1 (5%)	0	0	0	0	21 (95%)
AT	Discontinued Asp 128/1158 (11%)	7.7 (5.9, 9.9)	16 (13%)	2 (2%)	1 (1%)	5 (4%)	3 (2%)	103 (80%)
	Discontinued P 92/1158 (8%)	7.8 (6.0, 9.6)	7 (8%)	3 (3%)	2 (2%)	5 (5%)	3 (3%)	74 (80%)
	Discontinued AP 50/1158 (4%)	7.2 (6.1, 8.7)	5 (10%)	2 (4%)	2 (4%)	3 (6%)	2 (4%)	38 (76%)
	Initiated a different P2Y12 inhibitor 84/1158 (7%)	3.3 (1.7, 6.8)	8 (10%)	5 (6%)	0	1 (1%)	2 (2%)	68 (76%)

Follow-up was censored at time of first bleed; therefore, any patients who switched because of a bleed were not included in the analysis after the switch.

*MI or stroke. NB: ‘After switch’ includes switches on the same day as the event; for those who discontinued aspirin and clopidogrel, the earliest date of cessation was used.

AC, aspirin and clopidogrel; ACS, acute coronary syndrome; AP, aspirin and prasugrel; Asp, aspirin; AT, aspirin and ticagrelor; STEMI, ST-elevation myocardial infarction.

## Discussion

The main findings from this study based on a real-world English ACS population undergoing PCI are that: more potent DAPT (with prasugrel or ticagrelor) increases the risk of bleeding when compared with less potent DAPT (with clopidogrel); more potent DAPT does not decrease ischaemic or major adverse cardiovascular endpoints; the rate of overall bleeding was similar between DAPT with prasugrel and DAPT with ticagrelor; and adherence to the potent DAPT regimens prescribed at baseline was lower than adherence observed in RCTs.

The incidence of bleeding we observed in our study (9% in ACS and 10% in STEMI) is consistent with incidences reported in RCTs and observational studies (11%).^21 22^ Despite the fact that we had detailed information on the type of bleeds, we could not categorise bleeding severity due to the absence of laboratory parameters. However, it is reasonable to assume that CPRD bleeding is less severe since it was represented more frequently by skin or soft tissue bleeds and was more frequent than HES bleeding for both ACS (7% vs 2.5%, respectively) and STEMI (8% vs 3%, respectively).

In our study, ACS and STEMI treatment with ticagrelor was associated with approximately 50% increased risk of overall bleeding. In ACS, ticagrelor had a greater impact on CPRD than HES bleeding (60% and 33% higher, respectively). IN STEMI, ticagrelor doubled the risk of HES bleeding and increased risk of CPRD bleeding by 53%. Our results reflect those from recent meta-analyses^21 22^ (>25 000 patients) and a network meta-analysis in ACS populations revascularised by PCI (>52 000 patients),^23^ which showed increased risks of both major and minor bleeding (between 27% and 57%).

In STEMI, bleeding events were similar between prasugrel and ticagrelor (11% vs 12%, respectively). This confirms findings from other reports; the ISAR-REACT 5 trial,^24^ which included 4018 participants with ACS undergoing PCI, and the network meta-analysis above^23^ both showed no difference between prasugrel and ticagrelor for major bleeding (HR: 1.12, 95% CI 0.83 to 1.51 and HR: 0.99, 95% CI 0.79 to 1.24, respectively) or minor bleeding (HR: 0.90, 95% CI 0.76 to 1.06, ISAR-REACT 5).

In our study, ticagrelor and prasugrel did not reduce the risk of death or MACCE or any of the individual components of the MACCE composite compared with clopidogrel. Our findings reflect those from meta-analyses,^21 22^ which showed no significant association between ticagrelor versus clopidogrel and major adverse cardiovascular events (MACE) (OR: 0.83, 95% CI 0.66 to 1.03 and OR: 0.64, 95% CI: 0.41 to 1.01). Two network meta-analyses,^23 25^ including between 50 000 participants and 145 000 participants, show conflicting results. One failed to show a difference in MACE at 1 year between clopidogrel and prasugrel (OR: 0.81, 95% CI 0.60 to 1.11) or ticagrelor (OR: 0.82, 95% CI 0.60 to 1.10).^25^ The other showed that compared with clopidogrel, ticagrelor reduced cardiovascular (HR: 0.82, 95% CI, 0.72 to 0.92) and all-cause mortality (HR: 0.83, 95% CI 0.75 to 0.92) but not MI (HR: 0.97, 95% CI 0.78 to 1.22), whereas prasugrel did not reduce either cardiovascular or all-cause mortality (HR: 0.90, 95% CI 0.80 to 1.01 and HR: 0.92, 95% CI 0.84 to 1.02, respectively) but reduced MI (HR: 0.81, 95% CI 0.67 to 0.98).^23^

This inconsistency highlights the uncertain benefit of potent P2Y12 inhibition. Further evidence from the TOPIC trial^26^ showed a significant reduction in bleeding with no adverse impact on MACE, through switching from potent P2Y12 inhibition to clopidogrel, 1 month following ACS. Importantly, a difference in non-adherence was observed, with 25% of patients on unchanged DAPT regimen versus 14% of the clopidogrel switch group (p<0.01) not adhering to treatment. In our study, non-adherence was high across all three DAPT regimens (28% for clopidogrel and 31% and 33% for prasugrel and ticagrelor, respectively). This far exceeds the rate of observed in the PLATO trial (17%).^3^ Up to one-fifth of patients in all DAPT groups switched their first DAPT prescription, with median time to switch about 8 months in all groups. It is unclear to what extent non-adherence/switching influenced the findings with regards to bleeding or ischaemic outcomes in our tRCT.

### Strengths and limitations of this study

We used a real-world population and our datasets provided high resolution at the patient level with detection of bleeding of both major and minor severity and a thorough assessment of comorbidity, far extending the findings with regards to bleeding previously achieved through registry datasets.^27^ We used the tRCT approach as there is growing evidence that observational studies explicitly emulating existing RCTs can result in similar effect estimates to the RCT they are emulating,^28^ avoiding contradictory directions of effect.^29^ We identified confounders systematically using different sources.^30^

Our tRCT may be affected by residual confounding and selection bias. Patients assigned clopidogrel were older and had more comorbidities than patients assigned prasugrel/ticagrelor. Although these factors were adjusted for in the analyses, there remains the possibility that the groups still had different underlying risks of bleeding and ischaemia. We had no data on some confounders, for example, PCI procedural characteristics or severity of underlying disease, although these factors are more likely to bias ischaemic rather than bleeding outcomes. We had to exclude some eligible patients from the analysis (10% of ACS and 11% of STEMI) because we could not assign them to an intervention group. Their exclusion may have biased results for both bleeding and ischaemic outcomes. Nevertheless, the fact that our effect estimates remained largely unchanged following adjustment for confounders and the sensitivity analyses, including the excluded populations suggests that our estimates are robust.

## Data Availability

No data are available.

## References

[1] Levine GN, Bates ER, Bittl JA, et al. 2016 ACC/AHA guideline focused update on duration of dual antiplatelet therapy in patients with coronary artery disease: a report of the American College of Cardiology/American heart association Task force on clinical practice guidelines: an update of the 2011 ACCF/AHA/SCAI guideline for percutaneous coronary intervention, 2011 ACCF/AHA guideline for coronary artery bypass graft surgery, 2012 ACC/AHA/ACP/AATS/PCNA/SCAI/STS guideline for the diagnosis and management of patients with stable ischemic heart disease, 2013 ACCF/AHA guideline for the management of ST-elevation myocardial infarction, 2014 AHA/ACC guideline for the management of patients with non-ST-elevation acute coronary syndromes, and 2014 ACC/AHA guideline on perioperative cardiovascular evaluation and management of patients undergoing noncardiac surgery. Circulation 2016;134:e123–55. 10.1161/CIR.0000000000000404 27026020

[2] Valgimigli M, Bueno H, Byrne RA, et al. 2017 ESC focused update on dual antiplatelet therapy in coronary artery disease developed in collaboration with EACTS: the task force for dual antiplatelet therapy in coronary artery disease of the European Society of cardiology (ESC) and of the European association for Cardio-Thoracic surgery (EACTS). Eur Heart J 2018;39:213–60. 10.1093/eurheartj/ehx419 28886622

[3] Wallentin L, Becker RC, Budaj A, et al. Ticagrelor versus clopidogrel in patients with acute coronary syndromes. N Engl J Med 2009;361:1045–57. 10.1056/NEJMoa0904327 19717846

[4] Wiviott SD, Braunwald E, McCabe CH, et al. Prasugrel versus clopidogrel in patients with acute coronary syndromes. N Engl J Med Overseas Ed 2007;357:2001–15. 10.1056/NEJMoa0706482 17982182

[5] Ismail N, Jordan KP, Kadam UT, et al. Bleeding after hospital discharge following acute coronary syndrome: incidence, types, timing, and predictors. J Am Heart Assoc 2019;8:e013679. 10.1161/JAHA.119.013679 31657257 PMC6898798

[6] Ismail N, Jordan KP, Rao S, et al. Incidence and prognostic impact of post discharge bleeding post acute coronary syndrome within an outpatient setting: a systematic review. BMJ Open 2019;9:e023337. 10.1136/bmjopen-2018-023337 PMC6398751 30787079

[7] Hernán MA, Robins JM. Using big data to emulate a target trial when a randomized trial is not available. Am J Epidemiol 2016;183:758–64. 10.1093/aje/kwv254 26994063 PMC4832051

[8] Sterne JA, Hernán MA, Reeves BC, et al. ROBINS-I: a tool for assessing risk of bias in non-randomised studies of interventions. BMJ 2016;355:i4919. 10.1136/bmj.i4919 27733354 PMC5062054

[9] Herrett E, Gallagher AM, Bhaskaran K, et al. Data resource profile: clinical practice research Datalink (CPRD). Int J Epidemiol 2015;44:827–36. 10.1093/ije/dyv098 26050254 PMC4521131

[10] Hospital episode statistics (Hes). Available: http://content.digital.nhs.uk/hes

[11] Pufulete M, Harris J, Sterne JAC, et al. Comprehensive ascertainment of bleeding in patients prescribed different combinations of dual antiplatelet therapy (DAPT) and triple therapy (TT) in the UK: study protocol for three population-based cohort studies emulating 'target trials' (the ADAPTT Study). BMJ Open 2019;9:e029388. 10.1136/bmjopen-2019-029388 PMC6561407 31167875

[12] Main C, Palmer S, Griffin S, et al. Clopidogrel used in combination with aspirin compared with aspirin alone in the treatment of non-ST-segment-elevation acute coronary syndromes: a systematic review and economic evaluation. Health Technol Assess 2004;8:iii–iv. xv-xvi, 1-141. 10.3310/hta8400 15461878

[13] Terpening C. An appraisal of dual antiplatelet therapy with clopidogrel and aspirin for prevention of cardiovascular events. J Am Board Fam Med 2009;22:51–6. 10.3122/jabfm.2009.01.070282 19124633

[14] Wiviott SD, Braunwald E, McCabe CH, et al. Prasugrel versus clopidogrel in patients with acute coronary syndromes. N Engl J Med 2007;357:2001–15. 10.1056/NEJMoa0706482 17982182

[15] Atkinson MD, Kennedy JI, John A, et al. Development of an algorithm for determining smoking status and behaviour over the life course from UK electronic primary care records. BMC Med Inform Decis Mak 2017;17:2. 10.1186/s12911-016-0400-6 28056955 PMC5217540

[16] Bhaskaran K, Forbes HJ, Douglas I, et al. Representativeness and optimal use of body mass index (BMI) in the UK clinical practice research Datalink (CPRD). BMJ Open 2013;3:e003389. 10.1136/bmjopen-2013-003389 PMC3773634 24038008

[17] Kaura A, Sterne JAC, Trickey A, et al. Invasive versus non-invasive management of older patients with non-ST elevation myocardial infarction (SENIOR-NSTEMI): a cohort study based on routine clinical data. Lancet 2020;396:623–34. 10.1016/S0140-6736(20)30930-2 32861307 PMC7456783

[18] Cole SR, Hernán MA. Adjusted survival curves with inverse probability weights. Comput Methods Programs Biomed 2004;75:45–9. 10.1016/j.cmpb.2003.10.004 15158046

[19] Charlson ME, Pompei P, Ales KL, et al. A new method of classifying prognostic comorbidity in longitudinal studies: development and validation. J Chronic Dis 1987;40:373–83. 10.1016/0021-9681(87)90171-8 3558716

[20] Peterson AM, Nau DP, Cramer JA, et al. A checklist for medication compliance and persistence studies using retrospective databases. Value Health 2007;10:3–12. 10.1111/j.1524-4733.2006.00139.x 17261111

[21] Fan Z-G, Zhang W-L, Xu B, et al. Comparisons between ticagrelor and clopidogrel following percutaneous coronary intervention in patients with acute coronary syndrome: a comprehensive meta-analysis. Drug Des Devel Ther 2019;13:719–30. 10.2147/DDDT.S196535 PMC6388955 30863011

[22] Guan W, Lu H, Yang K. Choosing between ticagrelor and clopidogrel following percutaneous coronary intervention: a systematic review and meta-analysis (2007-2017). Medicine 2018;97:e12978. 10.1097/MD.0000000000012978 30412125 PMC6221558

[23] Navarese EP, Khan SU, Kołodziejczak M, et al. Comparative Efficacy and Safety of Oral P2Y_12_ Inhibitors in Acute Coronary Syndrome: Network Meta-Analysis of 52 816 Patients From _12_ Randomized Trials. Circulation 2020;142:150–60. 10.1161/CIRCULATIONAHA.120.046786 32468837 PMC7489363

[24] Schüpke S, Neumann F-J, Menichelli M, et al. Ticagrelor or prasugrel in patients with acute coronary syndromes. N Engl J Med 2019;381:1524–34. 10.1056/NEJMoa1908973 31475799

[25] Baldetti L, Melillo F, Moroni F, et al. Meta-Analysis Comparing P2Y_12_ Inhibitors in Acute Coronary Syndrome. Am J Cardiol 2020;125:1815–22. 10.1016/j.amjcard.2020.03.019 32305225

[26] Cuisset T, Deharo P, Quilici J, et al. Benefit of switching dual antiplatelet therapy after acute coronary syndrome: the topic (timing of platelet inhibition after acute coronary syndrome) randomized study. Eur Heart J 2017;38:3070–8. 10.1093/eurheartj/ehx175 28510646

[27] Völz S, Petursson P, Odenstedt J, et al. Ticagrelor is not superior to clopidogrel in patients with acute coronary syndromes undergoing PCI: a report from Swedish coronary angiography and angioplasty registry. J Am Heart Assoc 2020;9:e015990. 10.1161/JAHA.119.015990 32662350 PMC7660716

[28] Admon AJ, Donnelly JP, Casey JD, et al. Emulating a novel clinical trial using existing observational data. predicting results of the prevent study. Ann Am Thorac Soc 2019;16:998–1007. 10.1513/AnnalsATS.201903-241OC 31038996 PMC6774748

[29] Danaei G, Rodríguez LAG, Cantero OF, et al. Observational data for comparative effectiveness research: an emulation of randomised trials of statins and primary prevention of coronary heart disease. Stat Methods Med Res 2013;22:70–96. 10.1177/0962280211403603 22016461 PMC3613145

[30] Reeves BC, Deeks JJ, Higgins JPT. Chapter 13: Including non-randomized studies. In: Higgins JPT, Green S, eds. Cochrane Handbook for systematic reviews of interventions. version 5.0.2. The Cochrane Collaboration, 2009.

