# Supplementary material for: Real-world bleeding in patients with acute coronary syndrome (ACS) undergoing percutaneous coronary intervention (PCI) and prescribed different combinations of dual antiplatelet therapy (DAPT) in England: a population-based cohort study emulating a ‘target trial’
Source: Open Heart. 2022 Aug 12;9(2):e001999. doi: 10.1136/openhrt-2022-001999 (PMC9379532; doi:10.1136/openhrt-2022-001999)

Online Supplementary Material

**Table of contents**	**Page number**
Diagnoses and procedure codes used to identify the populations	2
Supplementary Figure 1. Propensity scores distribution for the ACS population.	3
Supplementary Figure 2. Propensity scores distribution for the STEMI population.	4
Supplementary Table 1. Baseline characteristics of included and excluded participants	5
Supplementary Table 2. Rates of HES-recorded bleeding, CPRD-recorded bleeding and total bleeding (HES and CPRD) in included and excluded participants	7
Supplementary Table 3. Crude and adjusted hazard ratios for association of antiplatelet prescription (AC vs. AP and AC vs AT) sensitivity analyses for the primary outcome in ACS and STEMI-only patients (2012-2017)	8
Supplementary Figure 3. Kaplan-Meier curves displaying cumulative all cause and cardiovascular mortality, mortality from bleeding and ischaemic events according to intervention group.	9
Supplementary Figure 4. Kaplan-Meier curves displaying cumulative all cause and cardiovascular mortality, mortality from bleeding and ischaemic events according to intervention group in the STEMI-only population	10

Pufulete M, *et al*. *Open Heart* 2022; 9:e001999. doi: 10.1136/openhrt-2022-001999

Diagnoses and procedure codes used to identify the populations

Office of Population Censuses and Surveys (OPCS) procedure codes for PCI:

K49 Transluminal balloon angioplasty of coronary artery
K50 Other therapeutic transluminal operations on coronary artery
K75 Percutaneous transluminal balloon angioplasty and insertion of stent into coronary artery

International Classification of Diseases (ICD-10) codes for ACS:

I20.0 Unstable angina
I21 Acute myocardial infarction
I22 Subsequent myocardial infarction
I24.9 Acute ischaemic heart disease, unspecified

STEMI-only:
I21.0 Acute transmural myocardial infarction of anterior wall
I21.1 Acute transmural myocardial infarction of inferior wall
I21.2 Acute transmural myocardial infarction of other sites
I21.3 Acute transmural myocardial infarction of unspecified site
I22.0 Subsequent myocardial infarction of anterior wall
I22.1 Subsequent myocardial infarction of inferior wall
I22.8 Subsequent myocardial infarction of other sites

Pufulete M, *et al. Open Heart* 2022; 9:e001999. doi: 10.1136/openhrt-2022-001999

Supplementary Figure 1. Propensity scores distribution for the ACS population

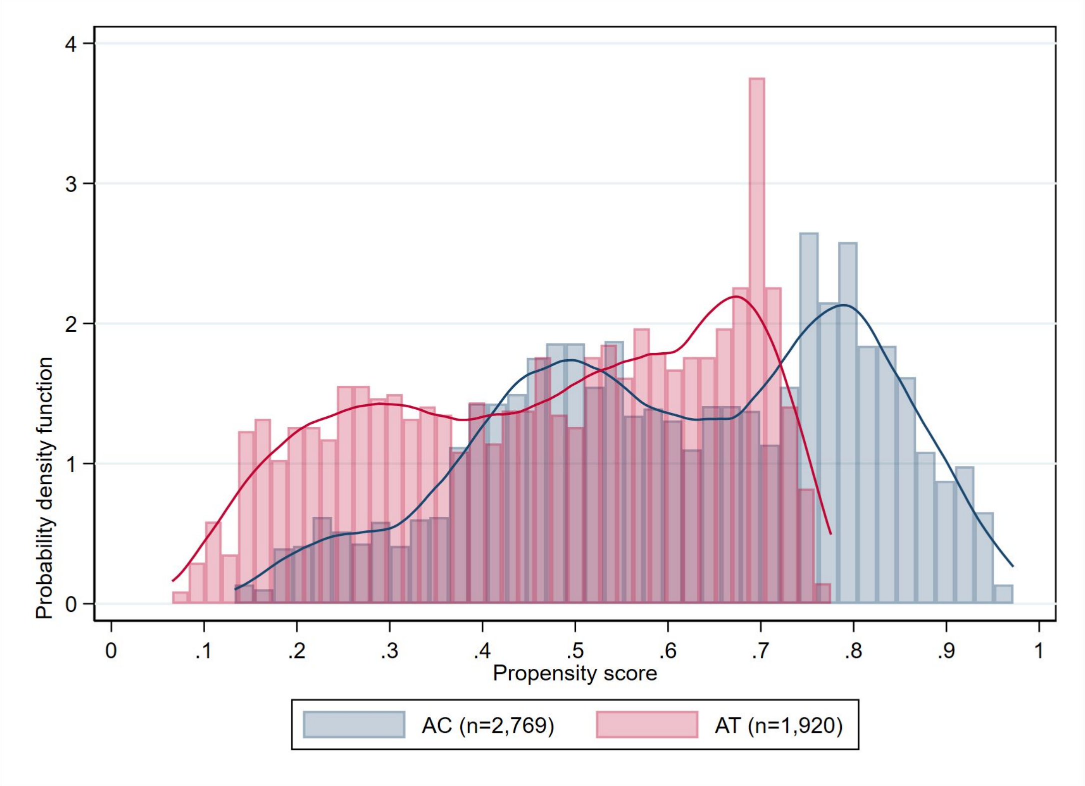

ACS: acute coronary syndrome; AC: aspirin and clopidogrel; AT: aspirin and ticagrelor

Pufulete M, *et al. Open Heart* 2022; 9:e001999. doi: 10.1136/openhrt-2022-001999

Supplementary Figure 2. Propensity scores distribution for the STEMI population

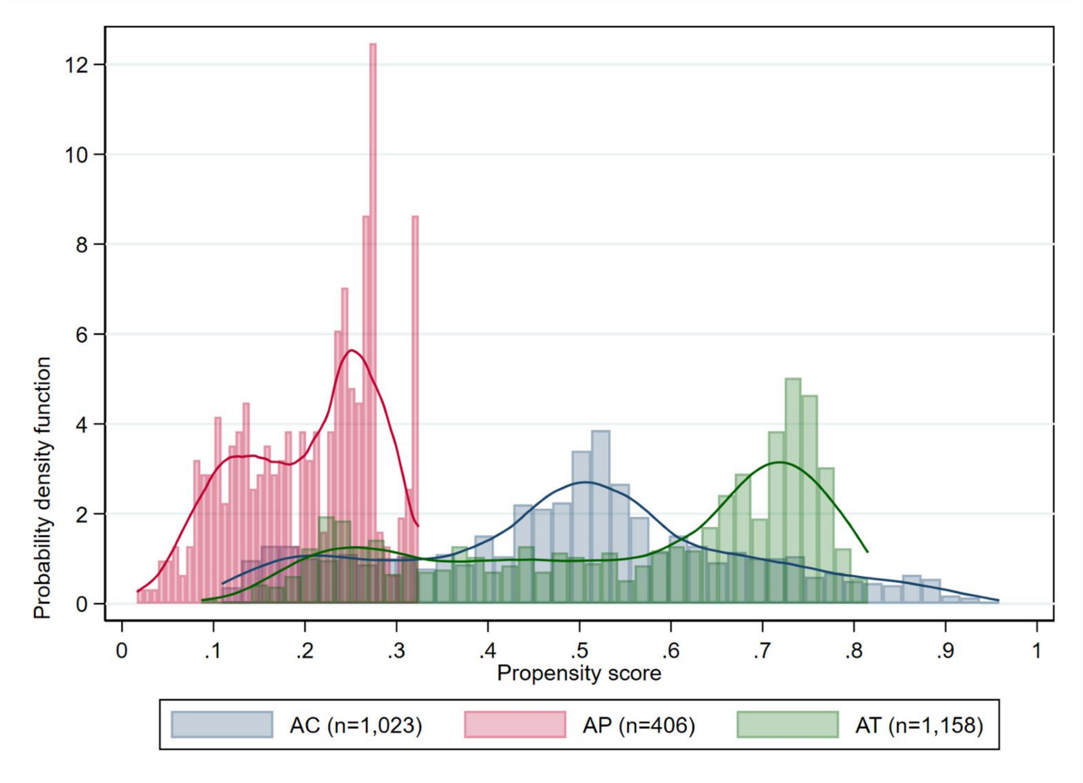

AC: aspirin and clopidogrel; AP: aspirin and prasugrel; AT: aspirin and prasugrel; STEMI: ST-elevation myocardial infarction

Supplementary Table 1. Baseline characteristics of included and excluded participants

		Acute coronary syndrome (ACS)					STEMI-only				
		Included n=4689	Ischaemic/ major bleed event or death before first prescription in CPRD n=250	SMD[1] (vs. Included)	No prescription in CPRD within 2 months of discharge n=270	SMD (vs Included)	Included n=2587	Ischaemic/ major bleed event or death before first prescriptio n in CPRD n=161	SMD (vs. Included)	No prescriptio n in CPRD within 2 months of discharge n=145	SMD (vs. Included)
Demography											
Year of event; n (%)	2012/13	1303 (28%)	67 (27%)	0.09	69 (26%)	0.11	713 (28%)	44 (27%)	0.09	40 (28%)	0.12
	2013/14	1147 (24%)	60 (24%)		64 (24%)		629 (24%)	35 (22%)		35 (24%)	
	2014/15	1025 (22%)	61 (24%)		57 (21%)		564 (22%)	39 (24%)		26 (18%)	
	2015/16	733 (16%)	41 (16%)		43 (16%)		423 (16%)	29 (18%)		27 (19%)	
	2016/17	481 (10%)	21 (8%)		37 (14%)		258 (10%)	14 (9%)		17 (12%)	
Age, yrs (mean; SD)		64.6 (12.4)	72.3 (12.9)	0.61	62.9 (13.6)	0.13	63.0 (12.3)	72.5 (13.7)	0.72	61.2 (13.3)	0.14
Sex; n (%)	Male	3418 (73%)	167 (67%)	0.13	207 (77%)	0.09	1923 (74%)	97 (60%)	0.30	116 (80%)	0.14
	Female	1271 (27%)	83 (33%)		63 (23%)		664 (26%)	64 (40%)		29 (20%)	
BMI[a], kgm^{-2} (mean; SD)		28.4 (5.2)	27.6 (5.3)	0.16	27.3 (5.2)	0.20	28.0 (4.9)	27.2 (4.9)	0.15	27.3 (4.9)	0.13
Ethnic group; n (%)	White	4275 (91%)	226 (90%)	0.03	244 (90%)	0.03	2382 (92%)	149 (93%)	0.02	132 (91%)	0.04
	Non-white	414 (9%)	24 (10%)		26 (10%)		205 (8%)	12 (7%)		13 (9%)	
Smoking category[b]; n (%)	Ex-smoker	1443 (32%)	92 (38%)	0.14	72 (28%)	0.18	722 (29%)	54 (36%)	0.16	37 (27%)	0.19
	Non-smoker	1699 (38%)	79 (33%)		83 (33%)		829 (34%)	52 (34%)		36 (27%)	
	Smoker	1357 (30%)	68 (28%)		98 (39%)		909 (37%)	46 (30%)		62 (46%)	
Medical History											
History of MI (ever); n (%)		3616 (77%)	149 (60%)	0.39	167 (62%)	0.34	1941 (75%)	88 (55%)	0.44	75 (52%)	0.50
History of CABG/PCI (ever); n (%)		1472 (31%)	93 (37%)	0.12	69 (26%)	0.13	593 (23%)	58 (36%)	0.29	29 (20%)	0.07
Bleeding; n (%)		92 (2%)	2 (1%)	0.10	8 (3%)	0.06	63 (2%)	-	0.22	6 (4%)	0.10
Previous surgery; n (%)		178 (4%)	17 (7%)	0.13	9 (3%)	0.02	75 (3%)	10 (6%)	0.16	2 (1%)	0.11
History of IHD (ever); n (%)		4084 (87%)	165 (66%)	0.51	187 (69%)	0.44	2052 (79%)	97 (60%)	0.42	77 (53%)	0.58
Diabetes; n (%)		924 (20%)	59 (24%)	0.09	33 (12%)	0.21	397 (15%)	32 (20%)	0.12	9 (6%)	0.30
Hypertension; n (%)		1857 (40%)	108 (43%)	0.07	78 (29%)	0.23	705 (27%)	69 (43%)	0.33	22 (15%)	0.30
Hypercholesterolaemia; n (%)		903 (19%)	34 (14%)	0.15	35 (13%)	0.17	263 (10%)	13 (8%)	0.07	13 (9%)	0.04

	Acute coronary syndrome (ACS)					STEMI-only				
	Included n=4689	Ischaemic/ major bleed event or death before first prescription in CPRD n=250	SMD[1] (vs. Included)	No prescription in CPRD within 2 months of discharge n=270	SMD (vs Included)	Included n=2587	Ischaemic/ major bleed event or death before first prescriptio n in CPRD n=161	SMD (vs. Included)	No prescriptio n in CPRD within 2 months of discharge n=145	SMD (vs. Included)
Peripheral vascular disease; n (%)	198 (4%)	15 (6%)	0.08	10 (4%)	0.03	72 (3%)	7 (4%)	0.08	8 (6%)	0.14
Stroke; n (%)	17 (0.4%)	3 (1%)	0.10	1 (0.4%)	0.001	11 (0.4%)	1 (1%)	0.03	1 (1%)	0.04
Heart failure; n (%)	308 (7%)	34 (14%)	0.24	24 (9%)	0.09	155 (6%)	20 (12%)	0.22	8 (6%)	0.02
Peptic ulcer disease; n (%)	14 (0.3%)	2 (1%)	0.07	0 (0%)	0.08	3 (0.1%)	0 (0%)	0.05	0 (0%)	0.05
Haemodialysis or renal disease; n (%)	196 (4%)	23 (9%)	0.20	8 (3%)	0.07	55 (2%)	12 (7%)	0.25	2 (1%)	0.06
Cancer; n (%)	191 (4%)	22 (9%)	0.19	17 (6%)	0.10	80 (3%)	14 (9%)	0.24	4 (3%)	0.02
Clotting disorder; n (%)	9 (0.2%)	1 (0.4%)	0.04	0 (0%)	0.06	5 (0.2%)	0 (0%)	0.06	0 (0%)	0.06
Anaemia; n (%)	106 (2%)	11 (4%)	0.12	8 (3%)	0.04	25 (1%)	3 (2%)	0.08	3 (2%)	0.09
Liver cirrhosis; n (%)	1 (0.02%)	2 (1%)	0.08	0 (0%)	0.02	0 (0%)	1 (1%)	-	0 (0%)	-
NSAIDs; n (%)	903 (19%)	40 (16%)	0.09	34 (13%)	0.18	450 (17%)	21 (13%)	0.12	13 (9%)	0.25
Steroids; n (%)	417 (9%)	34 (14%)	0.15	25 (9%)	0.01	209 (8%)	20 (12%)	0.14	11 (8%)	0.02
PPIs; n (%)	1612 (34%)	93 (37%)	0.06	65 (24%)	0.23	785 (30%)	54 (34%)	0.07	29 (20%)	0.24
Anticoagulants; n (%)	23 (0.5%)	7 (3%)	0.18	1 (0.4%)	0.02	11 (0.4%)	6 (4%)	0.23	0 (0%)	0.09

AC: aspirin and clopidogrel; AP: aspirin and prasugrel; AT: aspirin and ticagrelor; BMI: body mass index; CABG: coronary artery bypass graft; MI: myocardial infarction; NSAID: Nonsteroidal anti-inflammatory drugs; PCI: percutaneous coronary intervention; PPI: proton-pump inhibitor drugs. Missing data: [a] 407 patients; [b] 212 patients

[1] Restricted to 2012-2017

[2] DAPT is unknown for those who bleed before first prescription / further ACS before prescription / died before 2 months and no prescription / no DAPT in GP notes in first 2 months post-discharge (*see sensitivity analysis 1*)

[3] Unknown intervention (n=520) vs known intervention (n=5218)

Supplementary Table 2. Rates of HES-recorded bleeding, CPRD-recorded bleeding and total bleeding (HES and CPRD) in included and excluded participants

	Acute coronary syndrome (ACS)			STEMI-only		
	Ischaemic/ major bleed event or death before first prescription in CPRD n=250	No prescription in CPRD within 2 months of discharge n=270	Included n=4689	Ischaemic/ major bleed event or death before first prescription in CPRD n=161	No prescription in CPRD within 2 months of discharge n=145	Included n=2587
Any bleeding; n (%)	5 (2%)	9 (3%)	416 (9%)	3 (2%)	6 (4%)	260 (10%)
Major bleeding (HES-recorded); n (%)	6 (2%)	11 (4%)	117 (2%)	4 (2%)	5 (3%)	70 (3%)
Minor bleeding (CPRD-recorded); n (%)	1 (0%)	2 (1%)	332 (7%)	0	2 (1%)	208 (8%)
All-cause mortality; n (%)	219 (88%)	11 (4%)	104 (2%)	141 (88%)	2 (1%)	60 (2%)
Cardiovascular mortality; n (%)	195 (78%)	6 (2%)	48 (1%)	127 (79%)	1 (1%)	27 (1%)
Mortality from bleeding; n (%)	7 (3%)	0	7 (0.1%)	4 (2%)	0	6 (0.2%)
Myocardial infarction; n (%)	3 (1%)	9 (3%)	133 (3%)	2 (1%)	3 (2%)	72 (3%)
Stroke; n (%)	1 (0.4%)	1 (0.4%)	9 (0.2%)	1 (1%)	0	5 (0.2%)
Additional coronary intervention; n (%)	8 (3%)	29 (11%)	475 (10%)	6 (4%)	20 (14%)	293 (11%)
MACCE; n (%)	203 (81%)	40 (15%)	586 (12%)	133 (83%)	23 (16%)	350 (14%)

ACS: acute coronary syndrome; CPRD: Clinical Practice Research Datalink; HES: Hospital Episode Statistics; MACCE: major adverse cardiovascular and cerebrovascular events; STEMI: ST-elevation myocardial infarction.

Pufulete M, *et al. Open Heart* 2022; 9:e001999. doi: 10.1136/openhrt-2022-001999

Supplementary Table 3. Crude and adjusted hazard ratios for association of antiplatelet prescription (AC vs. AP and AC vs AT) sensitivity analyses for the primary outcome in ACS and STEMI-only patients (2012-2017)

	Acute coronary syndrome (ACS)				STEMI-only				
	All n=4689	Number of bleeds			All n=2587	Number of bleeds			
		AC n=2769	AT n=1920	HR (95% CI)		AC n=1023	AP n=406	AT n=1158	HR (95% CI)
Sensitivity analyses for primary outcome (any bleeding)									
Multiple imputation for unknown intervention group	5209*			1.45 (1.28, 2.39)[1]	2893*				AP: 1.77 (1.21, 2.58)[4]<br>AT: 1.47 (1.08, 2.00)[4]
Restricted to patients at low risk of bleeding; n (%)	390/4409 (8.8%)	196/2565 (7.6%)	194/1844 (10.5%)	1.44 (1.16, 1.80)[2]	248/2488 (10.0%)	78/982 (7.9%)	45/394 (11.4%)	125/1112 (11.2%)	AP: 1.76 (1.20, 2.58)[5]<br>AT: 1.46 (1.07, 2.00)[5]
Primary adjusted analysis without censoring of any CPRD or HES bleed at transfer out or last collection date; n (%)	427/4689 (9.7%)	215/2769 (7.8%)	212/1920 (11.0%)	1.46 (1.18, 1.80)[3]	265/2587 (10.2%)	81/1023 (7.9%)	47/406 (11.6%)	137/1158 (11.8%)	AP: 1.81 (1.24, 2.63)[6]<br>AT: 1.53 (1.12, 2.07)[6]

AC: aspirin and clopidogrel; CI: confidence interval; CPRD: Clinical Practice Research Datalink; HES: Hospital Episode Statistics; HR: hazard ratio; SA: sensitivity analysis. Models were adjusted for propensity scores (including year, age, gender, anticoagulants, ischaemic heart disease (ever), hypertension, myocardial infarction (ever), heart failure, STEMI, previous surgery [ACS]; including year, age, gender, myocardial infarction (ever), previous CABG/PCI, hypercholesterolaemia [STEMI]) and also adjusted for the following confounders which remained in the model after backwards elimination, these included: age, gender, BMI, ethnic group, smoking category, previous MI, previous CABG/PCI, previous bleed, previous surgery, previous ischaemic heart disease, diabetes, hypertension, hypercholesterolaemia, peripheral vascular disease, stroke, heart failure, peptic ulcer disease, haemodialysis or renal disease, cancer, clotting disorder, anaemia, liver cirrhosis, NSAIDs, steroids, PPIs, anticoagulants.

Supplementary Figure 3. Kaplan-Meier curves displaying cumulative all cause and cardiovascular mortality, mortality from bleeding and ischaemic events according to intervention group.

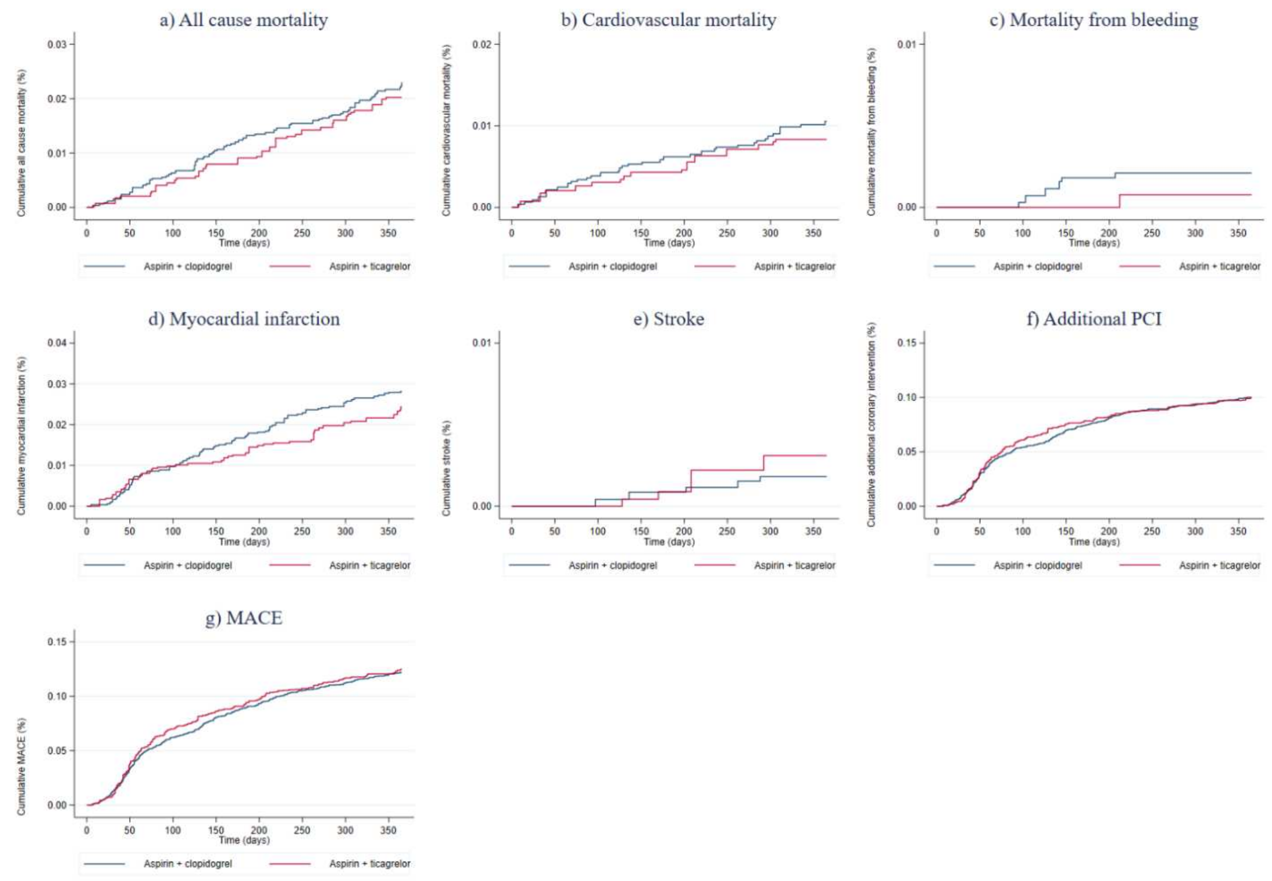

Supplementary Figure 4. Kaplan-Meier curves displaying cumulative all cause and cardiovascular mortality, mortality from bleeding and ischaemic events according to intervention group in the STEMI-only population

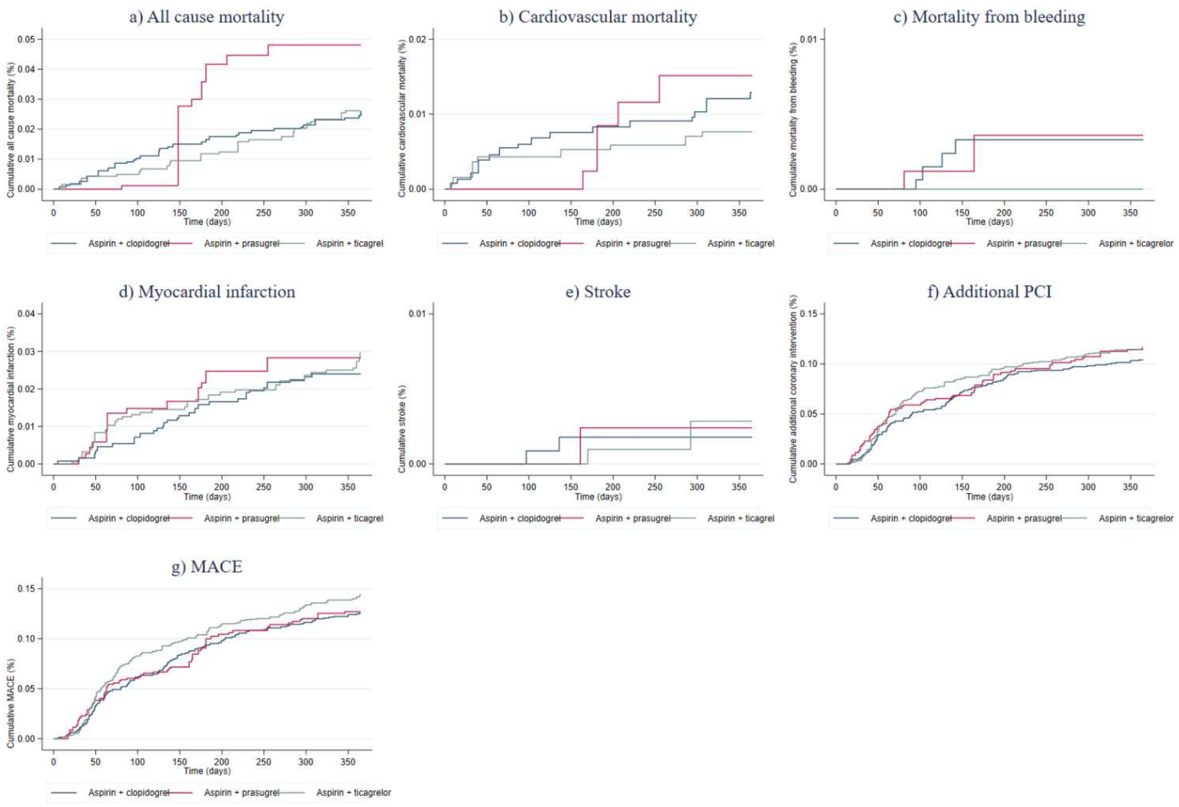

Supplement: Supplementary data [file openhrt-2022-001999supp001.pdf]
